# Methodological aspects of national surveys in Brazil: contributions to the debate on oral health surveillance

**DOI:** 10.1590/1807-3107bor-2025.vol39.043

**Published:** 2025-05-19

**Authors:** Andrea Maria Duarte VARGAS, Doralice Severo da Cruz TEIXEIRA, Maria Cecília Goi Porto ALVES, Gizelton Pereira ALENCAR, Regina Tomie Ivata BERNAL, Mara VASCONCELOS, Rafaela da Silveira PINTO, Andreia Maria Araújo DRUMMOND, Rosa Núbia Vieira de MOURA, Viviane Elisângela GOMES, Raquel Conceição FERREIRA

**Affiliations:** (a) Universidade Federal de Minas Gerais – UFMG, School of Dentistry, Departament of Social and Preventive Dentistry, Belo Horizonte, MG, Brazil.; (b) Ministério da Saúde, General Oral Health Coordination, Braília, DF, Brazil.; (c) São Paulo (Estado), Department of Health, Health Institute, São Paulo, SP, Brazil.; (d) Universidade de São Paulo – USP, School of Public Health, Department of Epidemiology, São Paulo, SP, Brazil.; (e) Universidade Federal de Minas Gerais – UFMG, Nursing School, Belo Horizonte, MG, Brazil.

**Keywords:** Oral Health, Health Surveys, Epidemiology, Public Health, Surveillance

## Abstract

This study analyzes the methodological aspects of the *SB Brasil* epidemiological surveys conducted in 2003, 2010, and 2023. The sample plan, fieldwork, sampling process, investigated variables, and operational aspects were examined based on technical documents and relevant publications. All three surveys adopted complex, probabilistic cluster sampling, with different study domains and sample sizes across editions. Data collection was conducted by SUS workers in all three editions, and Community Health Agents (ACS) were included as data collector in 2023. In this edition, the listing process occurred in a separate phase before the interview and examination, in two stages, to update the address list and identify eligible residents. Data collection included oral examinations and household interviews. The health conditions and oral health indices, as well as the criteria for defining the assessed conditions for each age group, remained similar across surveys. SB Brasil 2023 introduced, for the first time, the evaluation of the clinical consequences of untreated dental caries and the intervention urgency. There was an expansion of demographic and socioeconomic variables and subjective aspects of oral health over the editions. Increasing incorporation of technologies for data recording was observed, with automation of the sample selection process and report generation to support fieldwork monitoring. These changes reflect a continuous commitment to producing high-quality data, essential for supporting public policies and strengthening oral health surveillance in Brazil.

## Introduction

The National Oral Health Policy (PNSB), known as Brasil Sorridente, relies on epidemiological data on population health and disease conditions to guide oral health actions, aligned with the health surveillance model of the Brazilian Unified Health System (SUS).^1,2^ Since the 1980s, epidemiological surveys have been tools for public health planning. The 1986 national survey marked the beginning of this process by including children (6–12 years old), adolescents (15–19 years old), and adults (35–44 and 50–59 years old) from urban areas of 16 state capitals across Brazil’s five regions. Despite its limited geographic coverage and diversity of indicators, it served as a milestone for building a database that guided the first structured oral health interventions in Brazil. In 1996, the second survey expanded geographic coverage to the 27 state capitals but was restricted to examining dental caries in 6–12-year-old children from public and private schools.^3^ Despite these limitations, the survey reinforced the importance of epidemiological data and the need to broaden both the reference population and evaluated indicators. However, these surveys did not become an established component of the prevailing oral health policy.^4^

In 2003, the SB Brasil project was developed to produce information on the country’s status relative to the oral health targets for the year 2000 proposed by the World Health Organization (WHO). SB 2000 was based on the WHO methodology from the late 1990s^4^ regarding age groups and used dental index, employing a specific sampling design considering Brazil’s territorial complexity.^5,6^ This survey introduced a systematic methodological approach for sample collection and expanded evaluation of oral health indicators obtained through clinical examinations, as well as variables related to participants’ self-perceived health, service use, and sociodemographic characteristics.^6,7^ It marked a significant advancement, consolidating national surveys within the SUS.^8^

Subsequent surveys, SB Brasil 2010 and SB Brasil 2023, continued this model, maintaining a ten-year periodicity and incorporating methodological advancements based on accumulated experience.^8^ Moreover, these surveys consolidated the political decision to establish this strategy of obtaining primary data in the oral health surveillance axis as a core component of the SUS over time.^9,10^ The initiative has contributed to improving service evaluation and planning strategies while strengthening a methodological framework.

Over these decades, discussions have expanded on the need for methodological rigor to ensure reproducibility, validity, reliability, and uniform procedures for national and international comparisons^11^, as well as the challenges of conducting a national survey in Brazil, given its vast territory and significant heterogeneity across regions.^8^

These Brazilian experiences have resulted in a historical series, enabling an overview of oral health trends over time based on epidemiological data from the Brazilian population. To effectively serve its role in health surveillance, SB Brasil must ensure metric consistency, guaranteeing outcome comparability among its editions and with surveys conducted in other countries. In this context, the sustainability of national surveys, coupled with the necessary methodological care, ensures the continuity of a public policy oriented toward the real needs of the population and based on the oral health surveillance model. This study presents the main methodological aspects of SB Brasil 2023, highlighting comparisons with previous editions (SB Brasil 2003 and SB Brasil 2010). The objective is to understand methodological changes over time, indicating challenges in maintaining consistency within this oral health surveillance strategy in Brazil. Thus, this analysis aims to contribute to improving future editions and minimizing potential analytical inconsistencies when comparing variables over the years.

## Methods

In this document analysis,^12^ operational and methodological aspects of the last three population-based epidemiological surveys conducted in Brazil in 2003, 2010, and 2023, named SB Brasil 2003, SB Brasil 2010, and SB Brasil 2023, respectively, were described. Information about these surveys was obtained from publicly accessible documents or unpublished documents provided by researchers involved in the surveys.

For SB Brasil 2003, the consulted documents included Project SB2000 Oral Health Conditions of the Brazilian Population in the year 2000 – Examiner Manual^6^ and Coordinator manual^13^ and technical reports.^7,14^ For SB Brasil 2010, the consulted documents included Project SBBrasil 2010 Field Team Manual,^15^ Project SBBrasil 2010 Calibration Manual,^16^ Project SBBrasil 2010 Coordinator Manual,^17^ technical project, and technical report.^10^ For SB Brasil 2023, the technical project,^18^ technical report^9^ and Field Team Manual were consulted. Articles on the methodological aspects of SB Brasil 2003 and 2010.^8,19,20^ were also analyzed. Additional sources included WHO manuals for epidemiological oral health surveys (Oral Health Surveys Basic Methods) from 1987,^21^ 1997,^4^ and 2013,^22^ which served as methodological references for these surveys.

Information was described, comparing the three surveys in terms of sample design, investigated variables, field team composition and field work, and data recording methods. The study aimed to highlight aspects that remained consistent across all three editions. Data were synthesized textually and presented in comparative tables.

## Results

The three epidemiological surveys were conducted under the responsibility of the General Coordination of Oral Health at the Ministry of Health of Brazil, with different management and coordination models in each edition.^13,17^ The execution of SB Brasil 2023 was assigned to Universidade Federal de Minas Gerais (UFMG).

### Sampling plan

The sample design of all three surveys followed probabilistic sampling principles to obtain estimates for the reference population. This design evolved regarding reference population, sample size, and sampling process (Table 1). The urban population of Brazil was considered a reference in all three surveys, and, in 2003, the selection also included rural populations.^13^ All three surveys selected participants based on WHO-recommended index ages and age groups^4,22^ to assess oral health in children, adolescents, adults, and the older adults, with an adaptation to include adolescents aged 15–19 years. The decision to include the 15–19 age group considered that restricting selection to only 15-year-olds, as per WHO recommendations, would hinder fieldwork due to difficulties in enrolling adolescents. In 2003, an exploratory study was also conducted with children aged 18 months to 3 years. There were also differences in study domains, with regions and municipalities in 2003, state capitals and municipalities from the interior of the regions in 2010, and state capitals and Federative Units in 2023, impacting the respective sample sizes. The stages of selection also differed across the surveys, considering variations in the selection strata defined according to the estimation domains of interest.

**Table 1:** . Characteristics of the sampling plan in the oral health epidemiological surveys conducted in 2003, 2010, and 2023.

Sampling plan characteristics	SB Brasil 2003	SB Brasil 2010	SB Brasil 2023
Research population	Brazilian population from the six age groups included in the survey.	Brazilian residents in urban areas	Brazilian population residing in private permanent households in urban areas across the entire national territory in 2021
Sampling methods	Probabilistic sampling with stratification and clustering	Cluster probabilistic sampling	Cluster probabilistic sampling
Strata*	51 selection strata: 27 capital cities and 24 strata defined by the combination of region (five) with population size groups.	32 selection strata: Capitals of the 26 states, the Federal District, and 5 regions	53 selection strata:
	North: 5 strata		Federal District
	Northeast: 4 strata (for the >100,000 population stratum, no selection was made because, of the 10 cities selected, 9 were capitals, which already form a stratum)		26 state capitals
	Southeast: 5 strata		Municipalities in the interior of the 26 states
	South: 5 strata		
	Central-West: 5 strata		
Clusters**	Municipalities	Municipalities	Census tract
	Tracts	Census tract	
	Blocks/Rural villages		
	Schools or daycare centers		
Geographical domains ***	10 geographical domains: Five regions and municipalities according to population size	32 geographical domains: Capitals of the 26 states, the Federal District, and interior municipalities from each of the five Brazilian regions.	53 geographical domains, comprising the 26 state capitals and the Federal District, and the states as a whole (capital city and interior municipalities)
Demographic domains**	6 age domains	5 age domains	5 age domains
	18 months to 3 years	5 years	5 years
	5 years	12 years	12 years
	12 years	15 to 19 years	15 to 19 years
	15 to 19 years	35 to 44 years	35 to 44 years
	35 to 44 years	65 to 74 years	65 to 74 years
	65 to 74 years		
Stages of selection and sampling units	Municipalities with up to 50,000 inhabitants:	Capitals: 2 stages (census tracts and households)	5 and 12 years: 1 stage: census tract
	12 years: 3 stages (municipalities and educational establishments – schools, student)	Interior Municipalities: 3 stages (municipalities, tracts, and households)	Other age groups: 2 stages: census tract and households
	Other age groups: 3 stages (municipalities and urban blocks or rural villages and households) Municipalities with more than 50,000 inhabitants:		
	Capital cities (5 and 12 years): 2 stages: educational establishments – schools and daycare centers, student)		
	Capital (other age groups): 3 stages: tracts, blocks, and households		
	Other municipalities (5 and 12 years): 3 stages (municipalities, school, students)		
	Other municipalities (other age groups): 4 stages (municipalities, tracts, blocks/rural villages, households)		
Number of Primary Sampling Units (PSUs)	50 municipalities in each of the 5 regions, with 10 municipalities in each population size stratum: total of 250 municipalities.	Capitals: 30 census tracts per capital	Capitals: 1,365 tracts, ranging from 30 to 78 tracts per capital
	Selection of 20 schools in each municipality for adolescents aged 12 years	Interior of regions: 30 municipalities from each region, and 2 census tracts in each municipality	Interior: 458 tracts, ranging from 30 to 78 tracts per capital
Total sample size calculated	Total sample: 127,939	Total Sample: 47,005	Total sample: 50,800
	North: 23,656	5 years: 8,000	5 years: 250 capital + 100 in interior municipalities of the state
	Northeast: 26,400	12 years: 8,000	12 years: 250 capital + 100 in interior municipalities of the state
	Central-West: 24,255	15 to 19 years: 7,012	15 to 19 years: 300 capital + 100 in interior municipalities of the state
	Southeast: 28,660	35 to 44 years: 15,993	35 to 44 years: 300 capital + 100 in interior municipalities of the state
	South: 24,969.	65 to 74 years: 8,000	65 to 74 years: 300 capital + 100 in interior municipalities of the state
Sample size calculation	It was based on the average DMFT and standard deviation obtained in surveys from 1986 and 1996 and was calculated for each age and region and adjusted for finite populations for each population size stratum to obtain the sample for each municipality, based on the average number of people from that age group in each stratum. The total sample was calculated by multiplying the sample for each population size category by 10.	5, 12 and 65 to 74 years: The minimum number of interviews/examinations was determined based on the expectation that estimated prevalence rates would exceed 10% and that their standard errors would not surpass 15% (coefficient of variation) of these values.	The sample size was based on estimates of the mean DMFT and dmft indices obtained in SB Brasil 2010, as well as estimates of disease proportions close to 0.50. With these sample sizes, it was expected to estimate DMFT and dmft with a sampling error of <1 tooth (for 5- and 12-year-olds) and <1.3 teeth (for other age groups) and a CV < 20% for most estimates, except for those with proportions < 17%
		15 to 19 and 35 to 44 years: calculations were based on the variance of the mean DMFT observed in SB Brasil 2003.	
Selection of PSUs (1^st^ stage of the selection)	Municipalities: probability proportional to the population size of the region in the population size category. Capitals were included in the sample without a draw, within the category of municipalities with more than 100,000 inhabitants.	Capitals: Probability proportional to the number of permanent urban private households in each tract.	Capitals and interior: Census tracts were selected based on probability proportional to the number of private permanent urban households in each tract
		Interior Municipalities: Probability is proportional to the population size in each municipality	
Selection of sampling units in other sampling stages	Systematic selection (drawing) for blocks/rural villages	Households: Listing and selection were performed simultaneously, based on the definition of the sampling interval to be studied and identification on the listing sheet of households where sample elements from various age groups would be sought in municipalities based on the updated tract (2007). For other municipalities, where only the 2000 census data was available, a quick household count was conducted in the selected tracts, and this number was used to build the listing sheets.	Households: systematic selection after obtaining an updated list of households during the listing phase.
	Systematic selection of households in the selected blocks		
	Systematic selection of schools/daycare centers and students		
Obtaining estimates	Sample statistics were obtained. Sample weights were calculated *a posteriori* ^19^ for data weighting and sample estimate calculation.	The data was weighted by the inverse of the sampling fraction used, to compensate for differences in the probability of selection of the sampling units.	The data was weighted by the inverse of the sampling fraction used to compensate for differences in the probability of selection of the sampling units.

*Stratum: these are divisions of the population for which independent samples are selected. **Cluster: refers to a group of population units that is selected as a whole, rather than selecting individual units directly. Each cluster may be composed of, for example, individuals, households, schools, or municipalities. ***Domain: A subdivision of the population for which separate estimates are intended. The domain must have a minimum sample size.

### Fieldwork and sampling process

In all three surveys, a household data collection model was adopted, with differences in operational procedures.

In 2003, five-year-old children were selected from daycare centers in municipalities with > 50,000 inhabitants, while 12-year-old children were assessed in schools. In municipalities with ≤ 50,000 inhabitants, the local coordination team conducted the random selection of blocks, where selected households were visited to obtain the sample for the 5, 15–19, 35–44, and 65–74 age groups. In rural villages, households located within a 500-meter radius of a central point were visited. In larger municipalities (> 50,000 inhabitants), census tracts were randomly selected, followed by the random selection of blocks. Once the blocks were identified, the average number of households per block was calculated to determine the necessary total, and based on sampling intervals, the households to be visited were defined. In schools, after listing enrolled students, a systematic selection of participants^6^ was conducted (Table 1). In 2010, the household data collection model was based on the listing and identification of eligible households, considering the probability of finding residents from the target age groups. The field team conducted the listing and selection simultaneously, and once a resident was identified, the oral examination and interview were performed. It is worth noting that for census tracts without updated data on the number of permanent private households, this process was preceded by a rapid household count^8^ (Table 1).

In 2023, data collection was conducted in households through listing, interviews, and oral examinations, in three independent field visits involving an enumerator, an examiner, and a recorder. The listing, conducted in two phases, preceded the other steps. In Phase 1, the course of the selected census tract was based on maps of the census tract to identify buildings, while updating the addresses and the housing unit conditions (occupied, vacant, commercial etc.). This information was used to randomly select households for Phase 2, which aimed to identify potential participants in the index ages (5 and 12 years) and age groups (15–19, 35–44, 65–74 years). Subsequently, examiners and recorders returned to the households with eligible residents for interviews and oral examinations.^9^ The non-response rates were presented in technical reports from 2010 and 2023, with variations in the calculation methods.^9,10^ In 2010, the response rate was presented by domains and age groups, calculated by multiplying the ratio of surveyed households to the number of selected households by the ratio of completed examinations to surveyed households. Non-surveyed households included those that were vacant or whose residents refused participation. Non-completed examinations included cases categorized as not authorized, not permitted, or absence of the resident. In all age groups, the denominator for the first ratio was the target sample size. For instance, for the five-year-old group, 250 households were selected in all domains, although a larger number of households had to be visited to identify or confirm the presence of eligible children. In 2023, the report separately presented household and individual response rates for each age group in both capital and interior cities. The household response rate was defined as the ratio between the number of participating households (those that responded or provided information on the presence of residents in the target age group during the listing phase) and the total number of selected/visited households. Non-participating households included those whose residents refused or were unable to provide information on eligible residents, as well as those that were inaccessible after three or more attempts. Thus, the denominator varied for each age group and domain according to the number of identified and selected households in each census tract. The individual response rate was defined as the ratio between the number of invited individuals who participated (agreed to both the interview and examination) and the total number of eligible individuals identified in the selected households. Non-participating individuals included those who refused or were not found after three or more attempts. Therefore, the response rates reported in 2010 and 2023 are not comparable.

### Field team

In all three surveys, the field teams responsible for data collection were composed of professionals working in the SUS. In 2003 and 2010, each team consisted of a dentist (CD) and an oral health assistant (ASB). The CD served as the examiner, while the ASB acted as the recorder. In 2010, the coordinator’s manual indicated that, in exceptional situations where an ASB was unavailable, other professionals with a secondary or elementary education level could be part of the field team (as Community Health Agents (ACS)).^17^ In 2003, forming up to five field teams per municipality was recommended^13^. According to the technical report, approximately 2,000 workers from Municipal and State Health Departments participated in the study as examiners, recorders, or coordinators, having been temporarily released from their routine duties to conduct the study.^14^ In 2010, state capitals had 10 field teams, while municipalities in the interior had between 2 and 6 teams, depending on population size.^10^ In SB Brasil 2023, the team composition included a enumerator (list maker) preferably an ACS, in addition to the CD and ASB. The inclusion of Oral Health Technicians (TSB) in the teams was also allowed, with the ASB or TSB being able to act as either a recorder or an enumerator, depending on the availability of professionals in each context. The municipal coordination formed field teams, with the recommendation that one complete team be responsible for data collection in a maximum of three census tracts. In municipalities with only one selected census tract, a single team was allocated. Over time, changes in team composition and staff replacements occurred, totaling 637 CDs, 747 recorders, and 741 enumerators. All research professionals underwent training, and the examiners were calibrated to assess oral health conditions. The training and calibration steps for the examiners were detailed in the technical report.^9^ In 2023, training and calibration were conducted exclusively using the in-lux method for all conditions, and technological tools were employed for data recording and automated calculation of agreement coefficients. This process was reported in another publication within this same special volume.

### Data collection: investigated variables and data recording

Data collection in the three surveys was conducted through interviews with participants or parents/guardians and oral examinations. The manuals and technical projects indicated different levels of detail regarding who the respondents were during the interviews. In SB Brasil 2010, parents or guardians of 5-year-old children answered all interview questions. Similarly, in 2023, most questions were answered by the parents or guardians of 5-year-old children, but specific questions for the children themselves were included, and it was mentioned that an Assent to Participate Form was also obtained from the child. In 2010 and 2023, questions regarding the family’s socioeconomic condition were answered by the parents or guardians of 12-year-old adolescents. Questions about self-reported oral morbidity and self-perception of oral health were answered by the participants themselves in both surveys. In 2023, questions about the use of dental services by 12-year-old adolescents were answered by their parents or guardians, while participants aged 15-19 answered these questions themselves. In 2010, all these questions for these two age groups were answered by the participants themselves. In 2003, the examiner’s manual instructed that interview information should be “collected from the examined individuals only in the age groups of 15–19, 35–44, and 65–74 years.”

Information was collected on demographic and socioeconomic characteristics, self-reported oral morbidity, use of dental healthcare services, self-perception of oral health, and the impact of oral conditions on daily activities. Some questions remained unchanged across the surveys (Tables 2 to 4). Table 5 presents the number of questions in each survey, highlighting the higher number of questions in 2023 and the comparable questions among the surveys.^9,10^

**Table 2:** . Demographic and socioeconomic characteristics investigated in SB Brasil 2003, 2010, and 2023.

SB Brasil 2003	SB Brasil 2010	SB Brasil 2023
General information - Demographics		
Sex (male, female)	Sex (male, female)	Sex (male, female)
Age in years	Age in years	Age in years
Self-reported skin color or race according to IBGE*	Self-reported skin color or race according to IBGE*	Self-reported skin color or race according to IBGE*
Socioeconomic characteristics		
Number of people in the household	Number of people in the household	Number of people in the household
Number of rooms in the household	Number of rooms used as bedrooms in the household	Number of rooms used as bedrooms in the household
Car ownership (0 - Does not own a car, 1 - Owns a car 2 - Owns two or more cars)	Durable goods ownership (television, refrigerator, sound system, microwave, telephone, mobile phone, washing machine, dishwasher, personal computer, and number of cars)	Durable goods ownership (television, refrigerator, sound system, microwave, telephone, mobile phone, washing machine, dishwasher, personal computer, and number of cars)
Type of housing (Owned, owned with acquisition plan, Rented, Provided by others, Other)	x	x
x	x	Access to the internet via computer, tablet, mobile phone, television, or other devices
Family income (in BRL)	Family monthly income, including salaries, *Bolsa Família* (government benefit), pensions, rent, or other income (1: Up to 250; 2: From 251 to 500; 3: From 501 to 1,500; 4: From 1,501 to 2,500; 5: From 2,501 to 4,500; 6: From 4,501 to 9,500; 7: Over 9,500)	Family monthly income, including salaries, *Bolsa Família* (government benefit), pensions, rent, or other income (total amount in BRL)
Personal income (in BRL)	x	x
x	x	Receipt of Continuous Cash Benefit (BPC-LOAS) by a household member in the past year
x	x	Receipt of *Bolsa Família* by a household member in the past year
x	x	Receipt benefits from other government social programs by a household member in the past year.
x	x	Treated water supply in the household (1: Piped water in at least one room; 2: Piped water only on the property; 3: No piped water)
Student (yes or no)	x	Child attending preschool, early childhood education, daycare, or primary school (For 5-year-olds)
Type of School (Not a student, Public, or Private school	x	x
x	x	Child (or adolescent) can read and write (Yes/No)
x	x	Highest completed educational level, without failing (0 Did not attend school 1 Adult education 2 Incomplete Primary school 3 Completed Primary School 4 Incomplete Secondary School 5 Completed Secondary School 6 Incomplete Higher Education 7 Completed Higher Education)
		For children aged 5, the mother’s level of education was assessed
Schooling (years of study)	Total number of years studied with good performance (without failing).	Total number of years studied (Considering the completed level, grade, or school year without failing and excluding preschool, kindergarten, or daycare)

The cells highlighted in gray show measurements that are equal or similar across epidemiological surveys. *1 White; 2 Black; 3 Asian; 4 Mixed-race; 5 Indigenous

**Table 4:** . Characteristics related to self-perception of oral health, need for treatment, and impact of oral health on daily activities evaluated in SB Brasil 2003, 2010, and 2023.

SB Brasil 2003	SB Brasil 2010	SB Brasil 2023
Self-perceived oral health		
Classification of:	x	x
oral health		
appearance of teeth and gum		
chewing		
speech due to teeth and gums (Very Poor; Poor; Regular; Good; Excellent) (4 questions)		
How does oral health affect your relationship with others? (Not at all; A little; Moderately; Severely)	x	x
x	Satisfaction with oral health (Very satisfied; Satisfied; Neither satisfied nor dissatisfied; Dissatisfied; Very dissatisfied)	x
x	x	Self-perception of oral health (Very good; Good; Fair; Poor; Very poor)
Self-reported dental treatment needs		
Self-reported of need for dental treatment (No; Yes)	Self-reported of need for dental treatment ( No; Yes)	Self-reported of need for dental treatment (No; Yes)
x	x	Reason for self-reported dental treatment needs (Do not need dental treatment; Review/prevention/routine/cleaning; Gum bleeding; Toothache; Gum pain; Need braces; Need prosthesis (dentures, crowns, bridges, implants); Muscle or near ear pain; Need root canal; Need fillings (restorations); Bad breath; Tooth extraction; Whitening or other aesthetic treatment; Other
x	Self-perception of the need for full prosthesis (dentures) or to replace those currently used (No; Yes)*	Self-perception of the need for full prosthesis (dentures) or to replace those currently used (No; Yes)*
x	x	Presence of any implanted teeth or prosthesis in the mouth**
Oral impacts on daily performances		
x	Impact of oral health on daily activities:	Impact of oral health on daily activities:
	difficulty eating;	difficulty eating;
	speaking;	speaking;
	sleeping;	sleeping;
	discomfort brushing teeth;	discomfort brushing teeth; stopped practicing sports; teeth made you nervous or irritated;
	stopped practicing sports;	stopped going out, to parties, trips;
x	teeth made you nervous or irritated;	felt embarrassed to smile or talk;
	stopped going out, to parties, trips;	teeth interfered with studying/working or doing school/work tasks
	felt embarrassed to smile or talk;	(No; Yes)*** (9 questions)
	teeth interfered with studying/working or doing school/work tasks	
	(No; Yes)*** (9 questions)	
x	x	Self-Reported Scale of Oral Health Outcomes (SOHO-5) (parents)
		Has your child, because of their teeth, ever had difficulty eating; speaking; playing; sleeping; smiling due to appearance/aesthetics; stopped smiling because of holes in teeth, cavities, or tooth pain; and had their self-esteem/self-confidence affected (Not at all; A little; Somewhat; Significantly; A lot) ****
x	x	Self-Reported Scale of Oral Health Outcomes (SOHO-5) (children) Has it ever been difficult, because of teeth/tooth problems, to: eat; drink; speak; play; sleep; smile because you thought your teeth were ugly; smile because your teeth were hurting (No; A little; A lot) **** (faces scale)

*Self-perception of the need for dental prostheses assessed in the 15–19 years, 35–44 years, and 65–74 years age groups. **Presence of dental implants assessed in the 35-44 years and 65–74 years age groups. ***Impact of oral health on daily activities assessed in the 12 years, 15–19 years, 35–44 years, and 65–74 years age groups. ****Self-Reported Scale of Oral Health Outcomes assessed by parents or guardians for children aged 5 years and by the children themselves.

**Table 5:** . Number of questions in the interview with participants of SB Brasil in 2003, 2010, and 2023, and number of those that are comparable among surveys.

Aspects evaluated by interview	Total number of questions			Number of comparable questions			
	2003	2010	2023	All three surveys	Only 2003 and 2010	Only 2010 and 2023	Only 2003 and 2023
Demographic and socioeconomic characteristics	12	8	17	6	0	1	1
Self-reported oral health morbidity and use of dental services	6	6	11	5	0	1	0
Self-perception of oral health, treatment needs, and the impact of oral health on daily activities	6	12	19 (5 years) or 14 (12 years); 15–19, 35–44, 65–74 years	1	0	10	0

The variables related to demographic and socioeconomic characteristics common to all three surveys included: the number of people in the household, family income, participant’s sex, age in years, self-declared skin color or race according to the *Instituto Brasileiro de Geografia e Estatística* (IBGE), and education level (years of schooling). Regarding race/skin color, in 2003, for 5- and 12-year-olds examined in educational establishments, classification was conducted by the researcher based on predominant traits, according to biological characteristics described in the manual^6^. Concerning self-reported oral morbidity, the experience of toothache was assessed in all three surveys, though with different time frames: 3 months in 2003 and 6 months in the other two surveys. The severity of toothache was classified in 2003 as “mild,” “moderate,” and “severe pain,” while in 2010 and 2023, visual analog scales were used, with a range of 0 to 5 in 2003 and 0 to 10 in 2023. In 2023, for the first time, the report of orofacial pain was assessed in the age groups of 15–19, 35–44, and 65–74 years. The use of dental services was assessed based on time since the last dental visit, type of service used, reason for the visit, and evaluation of the service used in all three surveys, with some minor variations in response options (Table 2). In 2023, there was also an assessment of whether individuals sought and obtained dental care for all age groups. Regarding subjective aspects of oral health, the self-perception of the need for dental treatment was evaluated in all three surveys (Table 3). Notably, in 2023, the Self-reported Scale of Oral Health Outcomes, developed by Tsakos et al.^23^ and validated in Brazil by Abanto et al.,^24^ was used for the first time to assess the impact of oral conditions on daily activities among 5-year-old children, containing questions answered by both the children and their parents or guardians.

**Table 3:** . Characteristics related to self-reported oral morbidity and use of dental services evaluated in SB Brasil 2003, 2010, and 2023.

SB Brasil 2003	SB Brasil 2010	SB Brasil 2023
Self-reported oral morbidity		
How much pain the teeth and gums have caused in the last 3 months (0 - No Pain, 1 - Mild Pain, 2 - Moderate Pain, 3 - Severe Pain)	Reported dental pain in the last 6 months	Reported dental pain in the last 6 months
x	Pain severity of teeth (visual analog scale from 0 to 5 - 0 means no pain and 5 means very severe pain)	Pain severity of teeth (visual analog scale from 0 to 10 - 0 means no pain and 10 means very severe pain)
x	x	Reported orofacial pain in the last 6 months*
x	x	Pain severity of orofacial pain (visual analog scale from 0 to 10 - 0 means no pain and 10 means very severe pain)*
Use of dental services		
x	x	Access to dental healthcare services in the last year (sought a dental office, oral healthcare service, or dentist/oral healthcare team to be attended to) (Did not seek; Sought and was not attended to; Sought and was scheduled for another day/location; Sought and was attended to)
x	x	Type of dental office, oral healthcare service, or dentist/oral healthcare team sought (Did not seek; Public services; Private services; Health plan or insurance; Other)
x	x	Has a private dental plan, employer-sponsored or government-sponsored dental plan
Frequency of use of dental healthcare services (when was the last time you saw a dentist) (Never went to the dentist; Less than 1 year ago; 1 to 2 years ago; 3 or more years ago)	Frequency of use of dental healthcare services (when was the last time you saw a dentist) ( Never went to the dentist; Less than a year ago; 1 to 2 years ago; 3 or more years ago)	Frequency of use of dental healthcare services (when was the last time you saw a dentist) (Never went to the dentist; Up to a year ago; More than 1 year up to 2 years ago; More than 2 years up to 3 years ago; More than 3 years ago)
Type of dental healthcare service used at the last visit (Public services; Private practice; Private supplementary services (Health plans and insurance); Charitable services; Other)	Type of dental healthcare service used at the last visit (Public services; Private services; Health plan or insurance; Other)	Type of dental healthcare service used at the last visit (Never went to the dentist; Public services; Private services; Health plan or insurance; Other)
Reason for the last dental visit (Never went to the dentist; Routine checkup/repair/maintenance; Pain; Gum bleeding; Tooth cavities; Mouth sores, lumps, or spots; Swollen face; Other)	Reason for the last dental visit (Check-up, prevention, or maintenance; Pain; Extraction; Treatment; Other)	Reason for the last dental visit (Never went to the dentist; Cleaning, prevention, or check-up; Tooth pain; Extraction; Dental treatment (filling, root canal, etc.); Gum problems; Treatment of mouth sores; Dental implants; Placement/maintenance of braces; Placement/maintenance of prosthesis or dentures; Other)
Evaluation of dental healthcare services used (Evaluation of the last dental visit) (Never went to the dentist; Very Bad; Bad; Regular; Good; Excellent)	Evaluation of dental healthcare services used (Evaluation of the last dental visit) (Very good; Good; Regular; Bad; Very Bad)	Evaluation of dental healthcare services used (Evaluation of the last dental visit) (Never went to the dentist; Very Good; Good; Regular; Bad; Very Bad)
Received information on how to prevent oral problems	x	x

*The report of orofacial pain was assessed in the age groups of 15–19 years, 35–44 years, and 65–74 years.

In all three surveys, WHO methodological guidelines were used to define the conditions to be assessed and to conduct oral examinations.^4,22^ In 2003 and 2010, the 1987^21^ and 1997^4^ manuals were adopted, while in 2023, the 2013 version was also consulted.^22^ In 2000, a guide for epidemiological surveys produced by the USP School of Dentistry^25^ was also used, and in the 2010 and 2023, previous technical projects served as important references. The comparison of oral health conditions assessed in the three surveys is presented in Table 6.

**Table 6:** . Oral health conditions assessed in Brazilian epidemiological surveys in the years 2003, 2010, and 2023 for studied age groups.

Variable	2003							2010					2023				
	18–36 months	5	12		15-19	35-44	65-74	5	12	15-19	35-44	65-74	5	12	15-19	35-44	65-74
Dental condition																	
Permanent tooth crown	-	-						-					-				
Permanent tooth root	-	-	-		-			-	-	-			-	-			
Deciduous tooth crown			-		-	-	-		-	-	-	-		-	-	-	-
Need for treatment of deciduous and permanent teeth																	
Clinical consequence of untreated dental caries	-	-	-		-	-	-	-	-	-	-	-					
Dental fluorosis (Dean’s Index)	-				-	-	-	-	-		-	-	-	-	-	-	-
Dental Trauma	-	-	-		-	-	-										
Periodontal condition																	
Gingival alterations	-				-	-	-	-	-	-	-	-	-	-	-	-	-
Bleeding on probing	-	-	-		-	-	-	-					-				
Dental Calculus	-	-	-		-	-	-	-					-				
Shallow periodontal pocket (4 to 5 mm)	-	-	-		-	-	-	-	-				-	-			
Deep periodontal pocket (> 6 mm)	-	-	-		-	-	-	-	-				-	-			
Community Periodontal Index (CPI)	-	-						-					-				
Clinical Attachment Loss Index (Periodontal)	-	-	-		-			-	-	-			-	-	-		
Dentofacial anomalies (Dental occlusion)																	
Malocclusion index	-		-		-	-	-	-	-	-	-	-	-	-	-	-	-
Foster and Hamilton Index																	
Canine relation	-	-	-		-	-	-		-	-	-	-		-	-	-	-
Overbite	-	-	-		-	-	-		-	-	-	-		-	-	-	-
Overjet	-	-	-		-	-	-		-	-	-	-		-	-	-	-
Posterior Crossbite	-	-	-		-	-	-		-	-	-	-		-	-	-	-
Dental Aesthetic Index (DAI)	-				-	-	-	-			-	-				-	-
Use and needs for dental prosthesis																	
Use of dental prosthesis	-	-	-		-			-	-	-			-	-	-		
Need for dental prosthesis	-	-	-		-			-	-	-			-	-	-		
Other conditions																	
Intervention urgency	-	-	-		-	-	-	-	-	-	-	-					
Soft tissue changes								-	-	-	-	-	-	-	-	-	-

The green cells in the table indicate the oral health conditions assessed in each survey.

### Condition of dentition

The condition of dentition was assessed for all index ages and age groups, maintaining the same criteria across the three surveys. This record was made for each tooth separately, assigning codes according to the condition of the crowns of deciduous and permanent teeth: sound; decayed; filled, no caries; filled, with caries; missing due to caries; missing for other reasons; fissure sealant; fixed dental prosthesis abutment, special crown or veneer/implant placed for reasons other than dental caries; unerupted tooth or excluded tooth. In 2003 and 2010, in addition to these conditions, the presence of dental trauma was assessed and recorded as code T for all deciduous and permanent teeth. In 2023, this code was not recorded during the evaluation of the dental crown, following the 2013 WHO manual.^22^ The evaluation of dental conditions in the 35–44 and 65–74 age groups was complemented by the assessment of exposed roots, considering the same conditions except for missing teeth and sealant codes. There was also a change in 2023 regarding the records of deciduous dentition conditions. According to the WHO, since the 1997 manual, there have been no codes for recording deciduous teeth missing for other reasons and unerupted teeth. This guideline was followed in 2023, although in previous surveys, codes had been assigned for these conditions. In 1997, the WHO recommended the assessment of treatment needs for each tooth.^4^ However, in 2023, this index was maintained similarly to previous surveys, recording the need for restoration, dental crown, veneer for aesthetic reasons, pulp treatment, extraction, remineralization of white spot lesions, or pit and fissure sealants for deciduous and permanent teeth, despite the WHO having excluded this index in the 2013 manual.^22^ The evaluation of crown conditions enabled the calculation of the dmft and DMFT index, as recommended by the WHO, to estimate the experience of dental caries on deciduous and permanent dentition, respectively. In 2023, the assessment of clinical consequences of untreated caries (pufa/PUFA index) was included for all index ages and age groups, recording the presence of pulp involvement (P), ulceration (U), fistula (F), and dentoalveolar abscess (A) for each tooth with untreated caries.^26^

Dental trauma was assessed in all three surveys but with variations in the methods used. In 2003, code T was recorded for all deciduous and permanent teeth when there was a loss of part of the crown surface due to dental trauma, with no evidence of caries.^4^ In SB Brasil 2010, although code T was maintained, a specific index for assessing dental trauma in incisors at age 12 was introduced. The conditions assessed by the index were: no trauma, enamel fracture, enamel and dentin fracture, enamel and dentin fracture with pulp exposure, and tooth missing due to trauma. Following the trend anticipated in SB Brasil 2010, the 2013 WHO manual recommended assessing dental trauma through a specific index, in addition to removing code T from the evaluation of the crown condition. This guideline was followed in SB Brasil 2023, which, in addition to the conditions assessed in 2010, included the recording of trauma history (treated trauma) and other damages (lateral luxation, intrusion).

Dental fluorosis was assessed in 2003 (12 and 15–19 years old) and in 2010 (12 years old) using Dean’s Index, as recommended by the WHO,^4^ to record the severity of the condition in all teeth present in the mouth. This condition was not assessed in 2023.

### Periodontal condition

The assessment of periodontal condition followed the same criteria across the three surveys, using the Community Periodontal Index (CPI) to evaluate gingival bleeding, dental calculus, and periodontal pockets (shallow: 4–5 mm; deep: ≥ 6 mm) in the age groups of 15–19, 35–44, and 65–74 years. For 12-year-olds, only gingival bleeding and dental calculus were assessed. In addition to the CPI, clinical attachment loss was evaluated in the 35–44 and 65–74 age groups using the Clinical Attachment Loss (CAL) Index. Following WHO guidelines,^4^ both CPI and CAL assessments were conducted at least at six points on the buccal, lingual, mesial, or distal surfaces of index teeth in each sextant. In 2003, the worst CPI condition observed in each sextant was recorded. For example, if both calculus and periodontal pockets were present in the same sextant, only the periodontal pocket was recorded. In 2010, the presence or absence of gingival bleeding and dental calculus was recorded separately, along with the presence of shallow or deep periodontal pockets. In 2023, each condition was also recorded separately. For CAL, in all three surveys, the worst condition observed in each sextant was recorded. Additionally, in 2003, gingival bleeding was assessed during the dental crown examination in 5-year-old children. Those with three or more bleeding points during the examination were classified as having gingival bleeding. However, this assessment was not included in subsequent surveys.

### Dental occlusion condition (Dentofacial anomalies)

There were changes in the method used to assess dental occlusion in the deciduous dentition from 2010 compared to 2003. In 2003, the WHO-recommended malocclusion index^21^ was used, with modifications proposed by the USP School of Public Health, which classified children with normal occlusion, mild alteration, or moderate/severe alteration^4^. The last category was based on the evaluation of a combination of altered aspects: maxillary horizontal overjet > 9 mm, anterior crossbite > one tooth, open bite, and midline deviation. In 2010, the Foster and Hamilton^27^ index was used, which assesses canine relation, overjet, overbite, and posterior crossbite.^15^ The method adopted in 2010 was maintained in 2023.

For adolescents aged 12 and 15 to 19 years, the Dental Aesthetic Index (DAI), developed by Cons et al.^28^ and recommended by the WHO,^4^ was used to evaluate dentofacial anomalies. The DAI is based on a combination of 11 measurements, which express the individual’s occlusion status and need for treatment in three dimensions: (a) dentition – number of missing incisors, canines, and premolars in the upper and lower arches, (b) space (incisal segment crowding, incisal segment spacing, incisal diastema, maxillary anterior misalignment, mandibular anterior misalignment), and (c) occlusion itself (anterior maxillary overjet, anterior mandibular overjet, anterior vertical open bite, and anteroposterior molar relationship). These measurements generate a score calculated according to Jenny and Cons,^29^ to which cutoff points are applied to classify the individual as having normal occlusion or minor occlusal problems (score ≤ 25), defined malocclusion (score between 26 and 30), severe malocclusion (score between 31 and 35), and very severe malocclusion (score ≥ 36).

### Use and need for dental prosthesis

The evaluation of the use and need for prostheses was similar in all three surveys, following the criteria of the fourth edition of the WHO manual,^4^ with some modifications in 2010 and 2023 for the age groups of 15 to 19 years, 35 to 44 years, and 65 to 74 years. This evaluation considered the presence and size of the prosthetic space, as well as the type of prosthesis used to replace missing teeth: no prosthesis, one or more fixed bridge, removable partial prosthesis, a combination of fixed bridge and removable partial prosthesis, or removable full prosthesis. In 2023, a new code was introduced to record the use of fixed complete prostheses (overdenture). In addition to the prosthetic spaces, the need for a prosthesis also considered the quality of the removable partial or complete prostheses in use. An individual could be using a prosthesis but still require a new one if the current one is inadequate. The evaluation of quality was introduced in 2010 and maintained in 2023, based on the Prosthesis Quality Index.^30^ If the prosthesis had any issues in any of the following aspects – retention, stability, reciprocity, fixation, or aesthetics – it was considered unsatisfactory and needed to be replaced.

### Soft tissue alterations

This condition was assessed in 2003, considering the record of the absence or presence of any soft tissue alterations, without specifying the type or location of the lesion. Soft tissue evaluation was not maintained in subsequent surveys.^6^

### Intervention urgency

The evaluation of intervention urgency was conducted for the first time in the SB Brasil 2023 survey, following the guidelines of the 5^th^ edition of the WHO Manual.^22^ There is a need for immediate care (urgent) if pain, infection or serious illness is present or is likely to occur unless treatment is provided within a certain period of time. This period may vary from a few days to a month, depending on the availability of oral health services. Examples of conditions that require immediate attention are advanced dental caries, chronic alveolar abscesses, periapical abscess and acute necrotizing ulcerative gingivitis (ANUG). The need for referral for comprehensive evaluation or medical/dental treatment (systemic condition) was recorded when there were oral conditions related to life-threatening risks, such as oral cancer and pre-cancerous lesions, or any other suspicious lesion requiring evaluation/diagnosis for treatment. The needs for preventive or routine treatment or elective treatment were also recorded.

### Data recording

Table 7 presents the forms and methods used for data recording in the three epidemiological surveys. An evolution in the recording process was observed, shifting from a fully paper-based model (SB Brasil 2003), with subsequent data entry into specific software, to a hybrid system (SB Brasil 2010), which combined paper in the listing phase and electronic devices for data entry into specific software during the interview/examination. In the most recent edition (SB Brasil 2023), a fully digital method was adopted. Specific programs for data recording at each phase of the research were installed on Mobile Data Collection Devices, allowing direct field recording. Both in 2010 and 2023, data collection was performed offline, with data transmission occurring when internet access was available.

**Table 7:** . Forms, recorded information, and collected data recording methods in SB Brasil 2003, 2010, and 2023.

Epidemiological surveys	Forms for data recording	Recorded information	Recording method
SB Brasil 2003	Household Registration Form	- Census tract number (for municipalities > 50,000 inhabitants)	Paper sheets, no data entry performed
		- Block/Rural area number	
		- Household address	
		- Number of residents in the age groups studied	
		- Total number of households identified	
		- Total number of empty households or absent residents	
		- Number of residents identified in each age group	
		- Number of residents examined in each age group	
		- Number of residents who refused to participate in each age group	
		- Number of absent residents in each age group	
	Examination form	- Identification details (ID number, state, municipality, fluoride, years of fluoridation, census tract, block/neighborhood, school, examiner)	Paper sheets with information entered later into specific software for data tabulation for SB2000 in the municipality, if possible. Otherwise, the forms were sent to the state coordinators for data entry.
		- General information: Age, sex, ethnicity, age in months, geographic location, and examination performance	
		- Oral health conditions	
		- Socioeconomic characterization	
		- Access to dental services	
		- Self-reported oral health status	
SB Brasil 2010	Listing forms	- Region	Paper sheets prepared by the research coordination team were made available to guide fieldwork.
		- Municipality	
		- Census tract number	
		- Number of permanent private urban households	
		- Block	
		- Household location	
		- Indication of the demographic groups to be searched	
		- Space to enter the household status: occupied (participated, refused, closed), or vacant household	
	Data entry program for interview and oral examination data on an electronic device (Personal Digital Assistant – PDA)	- Identification (participant ID number, state, municipality, census tract, and household)	Software developed specifically for the survey. Response options for questions were pre-programmed and made available for selection during data entry. Data was recorded offline and sent to the coordinating team when internet access was available.
		- General information (age, sex, color or race)	
		- Record of examination performed or not (refusal, resident absence, or another reason)	
		- Interview (socioeconomic characterization of the family, education, reported oral morbidity and use of services, self-perception, and impacts on oral health)	
		- Oral examination (oral health condition indices)	
SB Brasil 2023	Data entry program for Phase 1 of household listing – updating the list of households in the selected tracts	A program pre-loaded with the IBGE (2019) street face databases for the randomly selected census tracts, so that the data recording for each block and face followed the tract’s route according to map indications:	- Data collected offline and sent to the coordinating team when internet access was available
		- Building Number	- The database was automatically generated and used as the basis for selecting households
		- Type of building (apartment-building, residential house, commercial, other buildings like schools, churches, etc., under construction or renovation)	
SB Brasil 2023	Data entry program for Phase 2 of household listing – identifying eligible participants in the selected households and tracts	A program pre-loaded with the list of households to be visited to identify residents within the target age groups, with information about the location of the households in the census tractor studied, including block number, face, and household number. For each household visited, the enumerator filled in:	- Data collected offline and sent to the coordinating team when internet access was available
		- Situation (whether or not the household has a participant of the target age, refusal to provide information about the resident’s age, not a household/household does not exist, vacant house – for sale, for rent, under construction, closed after 3 or more attempts)	- Automatically generated database used as a basis to provide the list of codes for residents to be interviewed and examined by a specific field team (access through a login and password for the team)
		- If a resident of the target age group is present, the number of residents was recorded, who will be the eligible participants	
	Data entry program for interview and oral examination data	A program pre-loaded with the list of codes of residents to be interviewed/examined, with information already linked to the identification of the household, block, street, and tract, so that the field team could return to the houses. The following records were made:	- Data collected offline and sent to the research coordination team when internet access was available. The questionnaire for recording interview and dental examination data was programmed with skips to minimize recording errors.
		- Socioeconomic and family characterization	
		- Participant’s or mother’s level of education	
		- Reported oral morbidity and access/use of oral healthcare services	
		- Oral health self-perception	
		- Impact of oral health on daily activities	
		- Oral health conditions	

## Discussion

The three editions of SB Brasil presented distinctions in the sampling plan, standardization of most of the oral health indices used, and an expansion in the number of interview questions, maintaining comparability in important sociodemographic and economic factors, use of services, self-reported morbidity, and self-perception of oral health variables. Advances were made in data recording methods, incorporating technological tools for the digital recording of interview and examination data (2010 and 2023), as well as data from the listing process (2023), and the use of a system for generating reports to monitor fieldwork (2023).

All three surveys used probabilistic sampling plans with cluster selection, aiming for precise estimates. However, differences in study domains limited temporal comparability to more aggregated levels, such as country and Brazilian regions. The three surveys defined different domains to reflect the country’s territorial diversity, on an increasing scale of logistical and methodological complexity. However, ensuring the accuracy of sample estimates across all domains is a challenge, especially for studying rare events. In 2003, the effort to obtain estimates at the municipal level involved a large sample size and longer data collection time. In 2010, two domains were chosen (state capitals and interior regions), allowing not only for estimates for these domains but also for evaluating indicators at regional and national levels. Nevertheless, some municipalities and states took the initiative to expand their samples to obtain estimates at their respective levels.^31^ In 2023, the sampling plan was changed again to obtain estimates for Brazilian states, in response to a demand from state leaders, increasing the sample size compared to 2010.^9^ This experience highlights the need to consider data production that supports the evaluation of policies, programs, and health decisions when designing sampling plans.

The oral health survey includes an assessment of various conditions, some with low prevalence in the population, which poses a challenge for sampling plans. The sample size would need to be significantly large to measure the frequencies of such events in all domains with adequate precision. After the survey results are released, changes in disease distribution and variability of measures over time are expected, due to the effects of public policies implemented. However, when using indicators collected more than a decade ago from previous surveys to calculate the sample size, the size may not be sufficient due to the expected reduction in the DMFT value as a result of decreasing caries, and the persistence of high variability due to ongoing inequalities. Therefore, it may be necessary for future surveys to expand the sample size to maintain the precision level of estimates by domain.

The sampling plan directly impacts the operationalization of fieldwork, particularly in the strategy for locating participants. In SB Brasil, all editions required creating a list of sampling units, though the strategies varied. In 2003, selection was based on blocks or rural villages, schools, or daycare centers, with lists prepared locally for selecting households or students. In 2010, the combination of strategies included a quick household count before the selection in tracts where demographic data was outdated, followed by the preparation of listing sheets by the central team, defining intervals for the selection. When household count data was updated, this initial step was skipped. In that year, listing occurred simultaneously with data collection. In 2023, due to the lack of updated household listings for the census tract, listing had to be done in advance to create an updated selection list. While this strategy aimed to better reflect the tract reality, it introduced a new fieldwork phase, increasing costs, professional staff requirements, and time. Using updated demographic census data could reduce fieldwork efforts and minimize errors from the additional listing phase.

The use of household surveys to research rare groups in the population, such as children, is also a challenge, as it involves the need to visit many households in the tract to locate people within the target age range. Considering the high enrollment rates in basic education in Brazil for children aged 4 to 5 (92.9%) and 11 to 14 years old (99.4%), according to PNAD 2023 data^32^, conducting surveys in schools may be more feasible, as recommended by the WHO for 5 and 12 years. The WHO also recommends including children aged 6 or 7 in countries where school enrollment occurs later, provided that the average age is reported in the results.^22^

Another challenge faced in population surveys is the high non-response rate. The difficulty in ensuring participation in health surveys has been widely discussed in the scientific literature, primarily due to the increasing challenges in contacting sample members and the rising number of refusals among those successfully reached.^33-38^ Various factors explain this phenomenon, including the method of managing the survey, question length, distrust in science, incentives, cultural differences, and lack of time to respond to interviews.^34,36,37,39^ In oral health surveys, the situation is even more challenging, as they include oral examinations, an essential stage in data collection, but one that requires considerable time due to the complexity of the conditions being assessed^35^. Many participants may refuse to take part, and in 2023, this difficulty was exacerbated by a post-pandemic COVID-19 context. Although the biosafety protocol, approved by ANVISA, was implemented and data collection started in 2022, the fear of contamination among the population remained. Additionally, the Brazilian political and social climate, marked by scientific denialism, rising violence, and distrust in research, worsened the situation. The difficulty attributed to increased violence rates was have been recognized in the literature.^40,41^ These factors resulted in participation refusals, difficulties in accessing housing complexes or buildings, and even the loss of entire census tracts. Even the demographic census, conducted in the country during the same period, faced similar refusal problems from the population.^42^

The literature points out several strategies to address the high non-response rate,^41^ many of which were implemented in 2023 with varying degrees of adherence by the field teams. Key strategies included community outreach (via letters, media campaigns, and health units), institutional support (such as transportation for high-risk areas), partnerships with local health units, meetings to engage municipal oral health managers, negotiations for field staff release, and flexible interviewer hours, including weekends. However, the last strategy faced challenges, as most field staff were restricted to regular hours, hindering participant identification at home. This underscores the need for continuous education to stress the importance of population surveys and professional roles within the SUS. Managers and professionals also faced difficulties reconciling survey demands with the backlog of clinical care from the two-year pandemic hiatus. High non-response rates can introduce bias, especially when non-respondents differ from respondents.^35,36,43^ Moreover, data analysis must account for the sampling plan, considering weights and aspects of the complex sampling plan, such as cluster sampling and stratification. To do this, analyses should be conducted using the survey modules of statistical software, which use the variables from the sampling plan, such as tract, stratum, and weights, to estimate the standard error. These methods were used in the 2010 and 2023 reports.^9,10^ In 2003, weight calculations were done later, allowing the use of data for historical comparisons of oral health conditions.^19,20^ The 2023 report described statistical methods, such as post-stratification weight adjustment, which were used to reduce biases potentially introduced by the underrepresentation of population groups. Outliers in the weight distribution were trimmed to minimize their impact on the standard error estimate and the design effect.^9^ It is noteworthy that the two most recent surveys reported non-response rates, although employing different methods for calculation. The literature has highlighted inconsistency in the way response rates are calculated and reported, if indeed they are reported at all.^35^. Survey response rate is a regarded as a key data-quality indicator, although response rate information alone is insufficient to determine the extent of non-nonresponse bias, or even whether it exists.^43,44^ However calculating these rates is a critical first step to understanding the presence of this potential survey error component.^45^

In all three surveys, the oral health conditions evaluated were defined with the clear intention of ensuring international comparability, adopting many of the WHO’s recommendations for epidemiological surveys. Additionally, in 2010 and 2023, efforts were made to maintain the historical series, such as the assessment of dental malocclusion through the DAI and the need for treatment, indices that, although no longer recommended by the WHO, were retained for their long-standing relevance. Another important criterion was the epidemiological relevance of the indicators. In this regard, in 2023, an index for assessing the clinical consequences of untreated dental caries (PUFA) was included, allowing the identification of disease severity in more affected groups, given the disease’s unequal distribution pattern in the population.^26^ On the other hand, the decision was made to discontinue the assessment of dental fluorosis, considering its endemic pattern in Brazil and the greater efficiency of addressing it in specific and targeted surveys. This decision was also supported by a study with data from SB Brasil 2003 and 2010, which discussed the wide variation in prevalence results observed between the years, the subjectivity in classifying fluorosis, difficulties in the calibration process, and the possible introduction of systematic error, as well as the impossibility of analyzing the trend of dental fluorosis in the national surveys of 2003 and 2010.^46^ As in 2010, in 2023, oral mucosa lesions were not assessed due to the technical inadequacy of cross-sectional population surveys for investigating these conditions, especially because of their rarity and the need for more appropriate methods for analyzing risk factors and incidence. In 2023, the separate recording of periodontal conditions was maintained, the WHO’s criteria for evaluating dental prostheses were adopted, and a specific index for dental trauma was included, as was done in 2010. However, concerning periodontal disease, the latest WHO manual recommends assessing and recording the condition of all teeth in the CPI, but this recommendation was not implemented due to the additional complexity it would add to the oral examination. The inclusion of intervention urgency evaluation provides a comprehensive view of the individual condition and helps estimate the prevalence of needs in the population based on the types of required care.

The inclusion of socioeconomic variables, subjective oral health variables, and variables related to the use of oral healthcare services in SB Brasil expanded the investigative power of the survey, enabling the analysis of various aspects of an individual’s life and understanding of health inequalities, as well as perceptions about oral health conditions and access to services. This approach aligns with the WHO’s recommendation to collect data on self-reported oral health and risk factors common to non-communicable chronic diseases.^40^ Socioeconomic variables, such as income and education, allow for analysis by subpopulations and monitoring of inequalities in oral health. Subjective indicators, such as self-perception of oral health, self-reported dental treatment needs and the impact of oral conditions on daily activities, complement the clinical evaluation^41^, reflecting functional aspects of oral health.^47^ Variables related to the use of services, such as the frequency of consultations, reasons for seeking care, and the type of service used, adds to our comprehension of the effectiveness of public policies and the barriers faced by the population. The integration of these data strengthens the analysis of needs and contributes to formulating more equitable interventions. However, greater advancement would be achieved by defining the variables supported by a conceptual model. Furthermore, it should be noted that cross-sectional studies are not suitable for investigating risk factors, as their design does not allow for the control of structural determinants, confounders, or effect mediators. Additionally, comparing information based on perceptions and reports is challenging, as it is subject to cultural influences, which may generate inconsistencies in the results across different countries.^40,41^

Oral health surveillance, defined as the systematic collection, analysis, and dissemination of data to prevent or control diseases and support public health actions,^48^ could be achieved with models that prioritize monitoring conditions of greater epidemiological relevance. Regional or municipal surveys can be conducted to explore specific issues or conditions, which can address management needs at the municipal or regional levels, often not covered by national research.^40,41^ In Brazil, other household-based surveys have been conducted by IBGE, such as the National Household Sample Survey (*Pesquisa Nacional por Amostra de Domicílios*), the National Health Survey (*Pesquisa Nacional de Saúde* – PNS),^41^ or school-based surveys, such as the National Student Health Survey (*Pesquisa Nacional de Saúde do Escolar* – PeNSE). The 2013 and 2019 PNS, and editions of PeNSE, included thematic modules on oral health, with self-reported oral health questions. In this sense, a potential strategy in the field of oral health surveillance is to strengthen the integration of oral health assessment within these institutionalized health surveillance practices.

Local research coordination and data collection were carried out by SUS workers in all three surveys and aimed at strengthening oral health surveillance actions and qualifying Primary Health Care teams. This choice was based on the expectation that training could promote greater autonomy in conducting oral health surveys more efficiently, as these professionals are already paid, saving time and resources compared to hiring and training external teams. Furthermore, this approach could empower professionals by integrating Epidemiology with managerial, clinical, preventive, educational, and health promotion aspects in oral health, both in routine services and dental knowledge.^31,49^ This orientation aligns with the guidelines of the National Oral Health Policy (PNSB), which recommends the use of epidemiological data on oral health to organize and plan oral health actions.^1,2^ It is also important to highlight that dentists, dental assistants, and dental hygienists have the participation in conducting surveys and epidemiological studies among their duties, as provided by the National Primary Care Policy.^50^ Additionally, conducting surveys is an action included in the Primary Care Service Portfolio.^51^ However, challenges have been noted, such as overload, demotivation, low involvement in training, the perception of research activities as extra duties, and dissatisfaction with the financial support provided. A study with members of the SB Brasil 2010 coordination team and with state and municipal oral health coordinators revealed that dissatisfaction with the amount and delay in payment was a demotivating factor, with 40% of participants stating that they would not want to participate again. The authors suggested that, as an activity linked to professional duties, dissatisfaction with the amount received could be related to discontent with public service remuneration^31^ and with the low incidence of these activities in their routine, given that surveys are not frequent. For future surveys, coordinators of SB Brasil 2010 suggested bonuses for teams with the best performance and the hiring of other professionals to conduct the examinations. In 2023, financial transfers were made to municipal and state oral health coordinators, as well as to field team professionals. Although the perception of these professionals was not assessed, it is assumed that the dissatisfaction felt in 2010 may have persisted in 2023. In 2023, ACS were included in the team as enumerators, considering their role characteristics and proximity to the work territory. This initiative was introduced in 2010, with ACS’s involvement assisting in the counting of households in tracts with outdated demographic data.^8^ The PNSB defines ACS’s duties as “Conducting demographic, social, cultural, environmental, epidemiological, and sanitary diagnosis of the territory in which they work, contributing to the process of territorialization and mapping the area of action of the team.”^50^ However, in SB Brasil 2023, in many locations, these professionals were unwilling to take on the task, as they did not recognize it as part of their duties and, in some cases, because they had not been released from their regular activities to perform this task. This problem highlights the limited integration of oral health with the Family Health Strategy (ESF). A previous study revealed that the joint development of actions, which require greater contact and engagement, is still incipient among teams. The Oral Health Team is primarily involved in clinical consultations, which limits work with the territory, family, and community in an integrated way with the ESF.^52,53^

An important advancement was the use of a data entry program during the data collection phase in 2010 and 2023. In 2010, a pioneering use of software for recording data collected through interviews and oral examinations was implemented in the national survey, resulting in significant gains, particularly in the security and quality of the data produced, as well as the speed with which the database was created.^8^ In 2023, for the first time, all data registration was digital, using a data entry program. This approach allowed the fieldwork to be optimized, as the data entry program was populated in advance, providing the necessary information for the teams to carry out data collection and recording directly on the mobile data collection device. In Phase 1 of the household listing, the program was configured with census tract databases. From the updated household listing and after automated random selections were made, the program was updated again, identifying each household to be visited by the field team to search for eligible residents in Phase 2 of the listing. This data was processed, and the list of households with residents of the interest age group was made available in the program so that the team could revisit the households for conducting interviews and examinations. Each phase involved detailed records of the field situation, both for the households and the residents. This process allowed the collection of information such as the number of visited households, presence of eligible residents, completion of examinations/interviews, refusals, and other situations. However, offline data collection brought challenges, as real-time field monitoring could not be performed, requiring data receipt and processing steps between research phases. This limitation sometimes led to delays and increased fieldwork duration. Nevertheless, the consolidated data, available in a fieldwork monitoring system, allowed researchers to track the progress of the survey by census tract, identifying problems and seeking solutions during the process. The incorporation of online data collection in future editions will represent a significant advancement and could become a reality with specific financial investment and the expansion of internet coverage across the country. This possibility was still limited in 2023, due to the presence of census tracts located in remote areas, often without internet access. Additionally, in 2023, logical skip features were incorporated into the interview and oral examination forms. Sequential questions, conditioned on previous answers, would not display or would only show the relevant options in the following questions. For example, if a participant reported not having used a service, the subsequent questions such as the type of service and the service evaluation would not appear. This programming may have contributed to reducing data entry errors during the data collection process.

Conducting such a complex survey requires significant human and financial investment, as well as a high level of commitment from oral health managers at national, state and municipal levels, especially for strengthening the oral health surveillance system. During the survey execution period, instabilities were observed in this area, as seven different professionals took on the role of the General Coordination of Oral Health, weakening negotiations with state and municipal managers. The continuity and improvement of oral health surveillance systems in Brazil depend on strategic investments. The accumulated lessons learned from the challenges faced, along with technological and methodological advances, can provide the foundations for strengthening oral health surveillance, ensuring the quality of oral healthcare services offered to the Brazilian population, as part of the guarantee of rights and towards social justice.

## Conclusion

The document analysis highlighted advancements in the execution of the surveys, with an increasing incorporation of technologies for data recording. There was standardization in many of the epidemiological variables and dental indices analyzed, with inclusions or removals justified by the epidemiological profile of disease distribution in the population. There was an expansion in the number of socioeconomic condition indicators and variables related to subjective aspects of oral health, revealing an effort to gain a more comprehensive understanding of disease distribution in the population, beyond normative aspects. The involvement of SUS professionals in all three editions of SB Brasil is based on the workers’ commitment to oral health surveillance actions. The changes reflect a commitment to producing high-quality data to support oral health public policies in Brazil. Despite the advancements, significant challenges remain, such as the need for greater uniformity in the variables investigated, increased engagement of field teams, and overcoming logistical and operational difficulties.
